# The Impact of Fasting the Holy Month of Ramadan on Colorectal Cancer Patients and Two Tumor Biomarkers: A Tertiary-Care Hospital Experience

**DOI:** 10.7759/cureus.33920

**Published:** 2023-01-18

**Authors:** Kanan Alshammari, Haifa A Alhaidal, Reem Alharbi, Alanood Alrubaiaan, Wesam Abdel-Razaq, Ghadah Alyousif, Mohammad Alkaiyat

**Affiliations:** 1 Oncology, King Abdulaziz Medical City Riyadh, Riyadh, SAU; 2 Pharmacy, King Saud Bin Abdulaziz University for Health Sciences, Riyadh, SAU; 3 Medicine, King Saud Bin Abdulaziz University for Health Sciences, Riyadh, SAU; 4 Pharmacology and Therapeutics, King Saud Bin Abdulaziz University for Health Sciences, Riyadh, SAU; 5 Pharmacy, King Abdulaziz Medical City Riyadh, Riyadh, SAU

**Keywords:** lactate dehydrogenase (ldh), carcinoembryonic antigen (cea), tumor biomarkers, colorectal cancer, intermittent fasting

## Abstract

Background: Fasting during the holy month of Ramadan is a religious ritual practiced by the majority of Muslims around the globe. This daytime fasting is short-term or intermittent fasting, which may be associated with valuable health benefits, particularly in cancer patients.

Methods: A prospective cohort study of pre- and post-fasting evaluation of 37 colorectal cancer (CRC) patients was conducted at King Abdulaziz Medical City (KAMC) and King Abdullah Specialized Children’s Hospital (KASCH)-oncology outpatient clinics. The study aimed to assess the impact of fasting during the holy month of Ramadan on the tolerability of chemotherapy side effects and to assess changes in the levels of carcinoembryonic antigen (CEA) and lactate dehydrogenase (LDH) tumor biomarkers, which are primarily associated with certain types of carcinomas, including CRC.

Results: A total of 33 patients (89.2%) had fasted at least part of the month of Ramadan. Twenty-seven patients (73%) reported “Serenity” after fasting during Ramadan with improved tolerability of chemotherapy side effects. However, the results did not reveal any significant difference in the measured laboratory variables between pre-fasting values and by the end of the 30 days of Ramadan. Although statistically insignificant, the levels of CEA and LDH were reduced in 46.9% and 55.6% of patients, respectively. The mean level of CEA in the fasting group was substantially reduced by more than 40%, attributed to the highly significant decline of CEA levels in three patients (p=0.0283). Moreover, there were no significant differences between pre- and post-fasting blood creatinine levels or estimated glomerular filtration rates, ruling out any possible adverse effects of fasting on renal function.

Conclusion: The current study confirms the safety and tolerability of intermittent fasting in CRC patients actively receiving chemotherapy, which is consistent with several reports. Nonetheless, the results did not reveal a significant decrease in CEA and LDH tumor biomarkers.

## Introduction

Daytime fasting during the month of Ramadan is a religious ritual practiced by the majority of Muslims around the globe. Ramadan is the ninth month of the lunar, also known as the Hijri calendar. Ramadan fasting is considered a distinctive model of intermittent fasting that refrains from eating and drinking all forms of foods, beverages, and even oral medications from sunrise to sunset each day until the end of the month of Ramadan, which may extend for 29 or 30 days depending on the visibility of the crescent moon. Such changes in the daily diet pattern may dramatically affect individual metabolic functions [1]. Several studies have reported valuable beneficial outcomes for intermittent fasting, including controlling obesity, energy metabolism, blood pressure, diabetes, cancer, and other diseases [2-6]. Although Muslims worldwide are fasting the holy month of Ramadan on an annual basis, the health consequences of such lifestyle practices are not adequately studied. Therefore, it is pivotal to evaluate the safety of this fasting practice in individuals with compromised health conditions, particularly cancer patients who are receiving cytotoxic chemotherapy.

In the Gulf countries, including Saudi Arabia, colorectal cancer (CRC) is still the topmost commonly diagnosed cancer in men and the third most common cancer, after breast and thyroid cancer, in women [7]. Although CRC is usually diagnosed in the elderly population, the incidence rate has peculiarly increased recently in younger adults under 50 years [8]. Several therapeutic modalities were employed in treating CRC patients, including surgery, radiation, and systemic chemotherapy, primarily depending on the cancer stage and patient’s factors. Chemotherapy regimens were shown to be favorably effective in advanced CRC stages [9]. However, cytotoxic chemotherapies have always been associated with numerous toxicities and adverse events. These include, but are not limited to, nausea and vomiting, myelosuppression, cardiotoxicity, renal impairment, fatigue, peripheral neuropathy, and others.

Quite a few studies have investigated the role of carcinoembryonic antigen (CEA) and lactate dehydrogenase (LDH) as prognostic tumor biomarkers in CRC patients [10,11]. High levels are usually associated with significant tumor burden due to the invasiveness and chemotherapy resistance in several malignancies, including CRC [12]. A recent study has employed the LDH-to-albumin ratio as a prognostic biomarker in CRC patients, which was associated with poor prognosis in CRC patients [13].

This study assessed the impact of fasting during the holy month of Ramadan on CRC patients concerning their tolerability of chemotherapy side effects, besides changes in blood parameters and levels of two tumor biomarkers (namely, CEA and LDH), which are primarily associated with several types of carcinomas, including CRC.

This article was previously posted to the medRxiv preprint server on August 6, 2022.

## Materials and methods

Study design

A prospective cohort study of pre- and post-medical and clinical evaluation of histologically confirmed CRC patients was conducted in the oncology outpatient clinics at King Abdulaziz Medical City (KAMC) and King Abdullah Specialized Children Hospital (KASCH), Riyadh, Saudi Arabia. The study protocol was reviewed and approved by the institutional review board of King Abdullah International Medical Research Center (KAIMRC), Riyadh, Saudi Arabia (#IRB/0846/21). Patients were identified from the hospital electronic information system called BESTCare® Software System, serving all entitled healthcare practitioners at different Ministry of National Guard Health Affairs (MNGHA) facilities. The population was limited to the available adult CRC patients on systemic therapy. Children less than 14 years and elderly patients with advanced and terminal diseases were excluded. The exclusion criteria also include pregnant, patients who had COVID-19, and patients whose primary oncologist recommended against fasting.

A total of 37 CRC patients were followed from the beginning of the month of Ramadan, corresponding to the Gregorian date (April 13, 2021), until the 20th of the next Hijri month of Shawal (corresponding to June 1, 2021). Patients’ demographic information including age, gender, weight, height, health status, and the received chemotherapies were retrieved from the unified electronic medical records. The current analysis considered baseline clinical data, including complete blood count, renal and liver functions, and serum level of two tumor biomarkers (CEA and LDH) measured immediately before the fasting month of Ramadan and by the end of the follow-up period.

Upon obtaining informed consent, all patients were asked to complete a questionnaire-based survey that explored their practice and attitudes toward fasting during the month of Ramadan, whether they complied with the healthcare provider’s recommendations and their subjective responses regarding tolerance of serious adverse effects during chemotherapy treatment.

Statistical analysis

Results are expressed as mean ± standard deviation (SD) and median with interquartile range (IQR) as dispersion characteristic for continuous data. Categorical variables are represented as proportions of total contributors. Graphs and statistical analyses were performed using GraphPad Prism® software package version 9.0 (San Diego, CA, USA). Statistical significance was considered at p-values less than 0.05 using unpaired student’s t-test between groups or Wilcoxon matched-pairs signed-rank test within the same group.

## Results

A total of 37 CRC patients’ data were reviewed. Table 1 displays the demographic and clinical characteristics of enrolled patients. The mean age was 52.7 ± 11.6 years, with a median value of 53 years (range 30-76). Almost half of the sample (45.9%) were overweight or obese patients with body mass indices of more than 25. Most of the sample had advanced stage III or IV carcinomas (27.0% or 67.6%, respectively). Most patients were treated with 5-fluorouracil-based chemotherapies (FOLFOX or FOLFIRI regimen, that included fluorouracil and leucovorin added to either oxaliplatin or irinotecan) as the standard of care treatment for advanced CRC. A bit more than 50% of patients also received a biological targeted therapy (cetuximab n=8, bevacizumab n=8, and panitumumab n=2) in addition to their primary standard cancer chemotherapy.

Patients were asked to complete a questionnaire survey exploring their fasting practice during the month of Ramadan (data not shown). About 92% of patients had consulted with their healthcare providers about the safety and appropriateness of fasting during the month of Ramadan, which coincided with their chemotherapy cycles. Almost all healthcare providers assured the safety of such intermittent fasting on CRC patients. Nearly one-third of patients had asked for dose rescheduling after sunset so that it would not affect their fasting practice during the daytime. In general, most patients (73%) reported “Serenity” after fasting during the holy month of Ramadan with perceived improved tolerability of chemotherapy side effects. About 8% of patients had unplanned visits to the emergency room during the month of Ramadan due to either pain or fever; one patient reported a skin rash. Less than 20% of responders reported feeling nauseous while fasting, but it did not affect their activities of daily living in most patients. This was confirmed by the Eastern Cooperative Oncology Group (ECOG) performance status scale, which determines patients’ ability to carry out their daily living activities during cancer chemotherapy and is reported by the treating physician (for details, see Table 1).

**Table 1:** General profile and cancer characteristics of enrolled CRC patients, n = 37 BMI: body mass index; CA: cancer; BSA: blood serum albumin; ECOG: Eastern Cooperative Oncology Group performance status

Variable	Value
Gender	n (%)
Male	17 (45.9%)
Female	20 (54.1%)
Age	In years
Mean ± (SD)	52.7 ± (11.6)
Median (range)	53 (30-76)
BMI	n (%)
< 18.5 (underweight)	4 (10.8%)
18.5-24.9 (healthy weight)	16 (43.2%)
25-29.9 (overweight)	9 (24.3%)
≥ 30 (obesity)	8 (21.6%)
Smoking	n (%)
No	35 (94.6%)
Yes	2 (5.4%)
Hypertension	n (%)
No	27 (73.0%)
Yes	10 (27.0%)
Hyperlipidemia	n (%)
No	30 (81.1%)
Yes	7 (18.9%)
Diabetes	n (%)
No	21 (56.8%)
Yes	16 (43.2%)
CA Family History	n (%)
No	33 (89.2%)
Yes	4 (10.8%)
ECOG Performance Status n (%)	n (%)
0	13 (35.1%)
1	22 (59.5%)
2	2 (5.4%)
3-5	0 (0.0%)
Tumor Site	n (%)
Colon	27 (73.0%)
Rectosigmoid	1 (2.7%)
Rectal	9 (24.3%)
Cancer Stage	n (%)
II	2 (5.4%)
III	10 (27.0%)
IV	25 (67.6%)

A total of 33 patients (89.2%) had fasted at least part of the month of Ramadan. On average, patients had fasted for 25 ± (5.3) days (range 10-30 days). Thus, it was decided to categorize patients into; Group A, which included patients who did not fast (n=4, 10.8%) or had fasted less than 20 days (n=7, 18.9%), and Group B, those patients who had fasted the entire 30 days of the month of Ramadan (n=13, 35.1%) or had fasted at least 20 days (n=13, 35.1%). Table 2 presents various demographic and clinical data of patients in both groups. The two groups show no significant difference, implying similar initial variables.

**Table 2:** Initial variables among the low-fasting and the high-fasting groups, n = 37 BMI: body mass index; CA: cancer; G-CSF: granulocyte colony-stimulating factor; BSA: blood serum albumin; ECOG: Eastern Cooperative Oncology Group performance status; eGFR: estimated glomerular filtration rate; CEA: carcinoembryonic antigen; LDH: lactate dehydrogenase. §Unpaired t-test

Variable	Group A None or fasted Less than 20 days (n=11)	Group B Fasted 20 days or more (n=26)	p-value^§^
Gender	n (%)		0.49
Male	6 (54.5%)	11 (42.3%)	
Female	5 (45.5%)	15 (57.7%)	
Age	In years		0.67
Mean ± (SD)	51.4 ± (11.7)	53.2 ± (11.8)	
Median (range)	57 (31-65)	51.5 (30-76)	
BMI	n (%)		0.78
< 18.5 (underweight)	2 (18.2%)	2 (7.7%)	
18.5-24.9 (healthy weight)	4 (36.4%)	12 (46.2%)	
25-29.9 (overweight)	3 (27.3%)	6 (23.1%)	
≥ 30 (obesity)	2 (18.2%)	6 (23.1%)	
Smoking	n (%)		0.52
No	10 (90.9%)	25 (96.2%)	
Yes	1 (9.1%)	1 (3.8%)	
Hypertension	n (%)		0.98
No	8 (72.7%)	19 (73.1%)	
Yes	3 (27.3%)	7 (26.9%)	
Hyperlipidemia	n (%)		0.32
No	10 (90.9%)	20 (76.9%)	
Yes	1 (9.1%)	6 (23.1%)	
Diabetes	n (%)		0.20
No	8 (72.7%)	13 (50.0%)	
Yes	3 (27.3%)	13 (50.0%)	
CA Family History	n (%)		0.83
No	10 (90.9%)	23 (88.5%)	
Yes	1 (9.1%)	3 (11.5%)	
G-CSF (Filgrastim)	n (%)		0.37
No	6 (54.5%)	10 (38.5%)	
Yes	5 (45.5%)	16 (61.5%)	
BSA	g/dL		0.41
Mean ± (SD)	1.8 ± (0.4)	1.7 ± (0.3)	
Median (range)	1.7 (1.2-2.3)	1.8 (1.2-2.2)	
ECOG Performance Status	n (%)		0.95
0	4 (36.4%)	9 (34.6%)	
1	7 (63.6%)	15 (57.7%)	
Hemoglobin (g/L)	g/L		0.29
Mean ± (SD)	121.1 ± (13.5)	115.0 ± (16.6)	
Median (range)	120 (105-150)	115.5 (88-147)	
White blood cells	x10^3^/mcL		0.20
Mean ± (SD)	6.0 ± (2.6)	6.2 ± (2.3)	
Median (range)	5.2 (2.5-11.7)	5.9 (1.7-11.8)	
Platelets	x10^3^/mcL		0.57
Mean ± (SD)	236.4 ± (149.4)	215.2 ± (76.5)	
Median (range)	175 (68-490)	207 (94-450)	
eGFR	mL/min/1.73m^2^		0.53
Mean ± (SD)	97.0 ± (36.9)	103.6 ± (25.5)	
Median (range)	109 (37-140)	103 (66-160)	
Alkaline phosphatase	IU/L		0.13
Mean ± (SD)	112.4 ± (42.7)	160.3 ± (98.0)	
Median (range) 44-147	96 (68-193)	121.5 (70-416)	
Missing data = 2			
CEA level	ng/mL		0.24
Mean ± (SD)	15.6 ± (32.4)	83.4 ± (184.1)	
Median (range) <3	2.9 (1.7-89)	12.1 (1.7-603)	
Missing data =5			
LDH level	mmol/L		0.23
Mean ± (SD)	230.9 ± (121.0)	299.8 ± (169.4)	
Median (range)	183 (129-487)	221.5 (154-689)	
Missing data =10			

**Figure 1:**
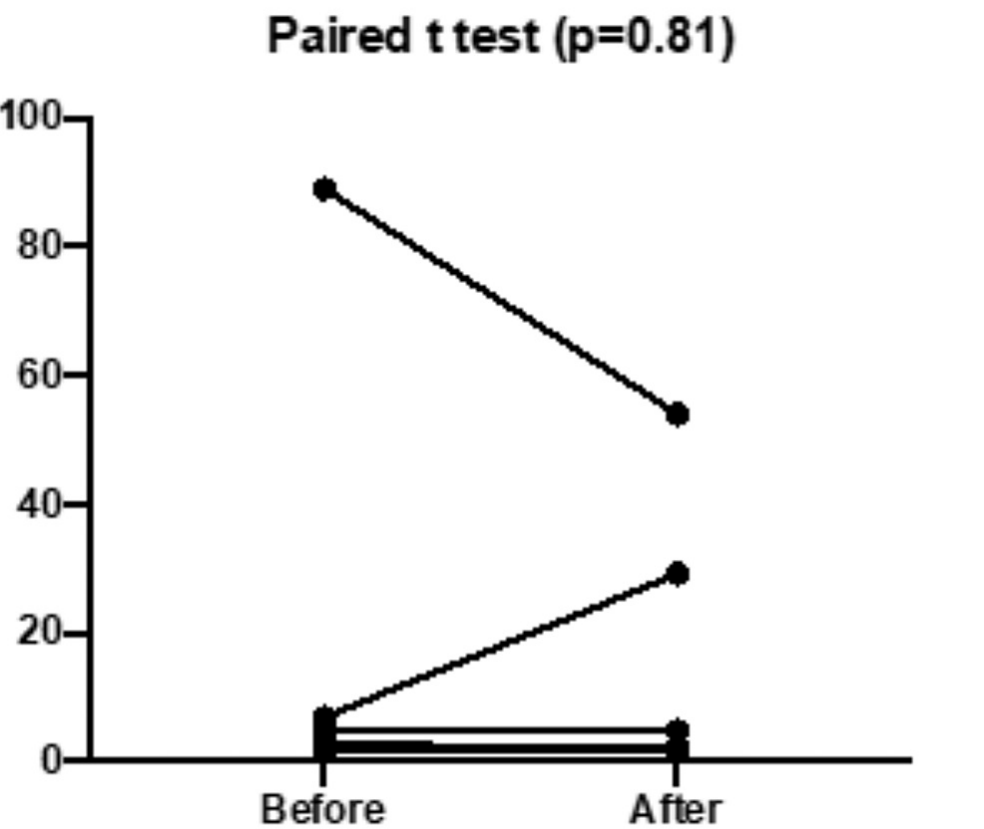
Carcinoembryonic antigen (CEA) levels (ng/mL) before versus by the end of the month of Ramadan in the low-fasting group (n=7).

**Figure 2:**
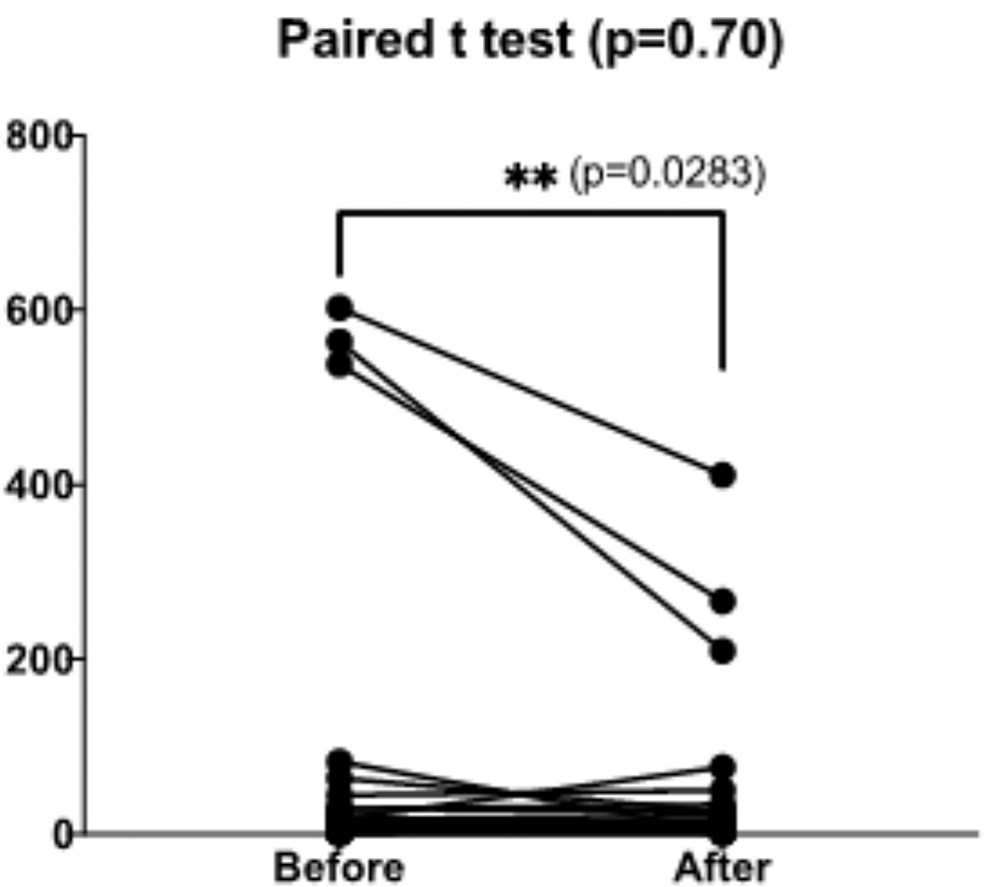
Carcinoembryonic antigen (CEA) levels (ng/mL) before versus by the end of the month of Ramadan in high-fasting group (n=25). CEA levels (ng/mL) before versus by the end of the month of Ramadan in the low-fasting (Group A) and the high-fasting (Group B). The p-value (0.0283) was calculated for the three samples that showed a significant reduction in CEA levels.

On the other hand, Table 3 shows the mean differences between initial variables and after fasting, if any, by the end of the 30 days of the month of Ramadan. The measured blood parameters were almost comparable. Although statistically insignificant, both tumor biomarkers, CEA and LDH, were reduced by 12.4% and 21.8%, respectively, in Group A; and declined by 40.9% and 15.5%, respectively, in Group B. Nevertheless, one sample in Group A showed a noteworthy decrease in CEA levels from 89 to 54 mmol/L, whereas another sample in Group A showed an increase in CEA levels from 6.9 to 29.2 mmol/L (Figures 1-2). Three samples in Group B showed momentous decreases in CEA levels from a mean value of 568.3 ± (32.7) to 296.0 ± (103.6) ng/mL with a highly significant p-value of 0.0283.

**Table 3:** Mean difference value analysis, n = 37 eGFR: estimated glomerular filtration rate; CEA: carcinoembryonic antigen; and LDH: lactate dehydrogenase. Advanced cancer stage IV was 63.6% and 69.2% in Groups A and B, respectively. § Calculated between means (Wilcoxon matched-pairs signed-rank test)

Fasting	Group A: None or fasted less than 20 days (n=11)				Group B: Fasted 20 days or more (n=26)			
Variable	Before Mean ± (SD)	After Mean ± (SD)	Mean Diff (%)	p-value*	Before Mean ± (SD)	After Mean ± (SD)	Mean Diff (%)	p-value^§^
Hemoglobin (g/L)	121.1 (13.5)	123.6 (12.0)	2.55 (2.1%)	0.27	115.0 (16.6)	115.5 (15.3)	0.46 (0.40%)	0.31
White blood cells (x10^3^/mcL)	6.0 (2.6)	7.4 (5.7)	1.38 (23.1%)	>0.99	6.2 (2.3)	6.6 (3.2)	0.38 (6.1%)	0.77
Platelets (x10^3^/mcL)	236.4 (149.4)	250.4 (159.4)	14.00 (5.9%)	0.17	215.2 (76.5)	235.7 (116.2)	20.54 (9.5%)	0.46
eGFR (mL/min/1.73 m^2^)	97.0 (36.9)	94.3 (32.0)	-2.73 (-2.8%)	0.71	103.6 (25.5)	104.3 (24.7)	0.73 (0.7%)	0.82
Alkaline Phosphatase (IU/L) Missing data =2	112.4 (42.7)	133.7 (65.4)	21.36 (19.0%)	0.20	160.3 (98.0)	164.6 (102.6)	4.38 (2.7%)	0.76
CEA level (ng/mL) Missing data =5	15.6 (32.4)	13.7 (20.4)	-1.93 (-12.4%)	0.81	83.4 (184.1)	49.3 (99.2)	-34.10 (-40.9%)	0.71
LDH level (mmol/L) Missing data =10	230.9 (121.0)	180.6 (40.7)	-50.29 (-21.8%)	0.44	299.8 (169.4)	252.7 (93.4)	-47.05 (-15.7%)	0.26

## Discussion

Despite the notable advances in cancer treatment, cancer remains a considerable burden on the healthcare system.

According to the American Society of Clinical Oncology and the European Society for Medical Oncology guidelines, regular screening of asymptomatic adults with an average risk is recommended at 50 years old [14,15]. This age has decreased recently to 45 years due to the steady increase of CRC among the young. However, there are few sensitive and specific tumor biomarkers for detection and/or assessing the efficiency of anticancer therapy. Currently, tumor biomarkers are rarely used to screen cancer either due to the absence of specific biomarkers or their inconsistent expression in cancer cells. Serum CEA is the most widely known diagnostic and prognostic tumor biomarker in recurrent CRC. LDH has also been utilized in the oncology practice as a biomarker of tumor progression. Both biomarkers are not specific for CRC only, as elevated levels of CEA and LDH can also be concurrent with other cancers and in several inflammatory and metabolic disorders [10,11]. Nevertheless, CEA remains a non-invasive tumor biomarker to monitor CRC progression and the efficacy of anticancer treatment.

The current study investigated changes in the blood parameters and levels of the two tumor biomarkers (CEA and LDH) due to intermittent fasting during the month of Ramadan in CRC patients receiving chemotherapy. The results did not reveal any significant difference in the measured variables between the initial values and by the end of the 30 days of the month of Ramadan. Although statistically insignificant, the levels of CEA and LDH were reduced in the majority of CRC patients. Decreased levels of these biomarkers can also be due to the chemotherapy used. However, the mean level of CEA in the fasting group was substantially reduced by more than 40% attributed to the highly significant decline of CEA levels in three patients (p=0.0283) who had remarkably extreme levels of CEA. Nonetheless, the high CEA levels were associated with poor survival in CRC patients [16]. Several studies have appraised the role of CEA tumor biomarkers as a diagnostic and prognostic tool, especially in the advanced CRC stages [10,17]. Others reported suboptimal accuracy of this tumor biomarker for CRC [18,19].

Results of the current study also revealed an insignificant decrease in the levels of LDH among CRC patients in both fasting and non-fasting groups. However, some studies reported a significant correlation between LDH levels and chemotherapy response as well as with the overall survival in patients with high LDH levels [20]. Another study reported significant benefits of bevacizumab as targeted therapy when combined with the standard of care treatment of metastatic and advanced CRC in patients with high LDH levels [21].

Therefore, all these results support the potential role of CEA and LDH levels as predictive tumor biomarkers in CRC patients. Moreover, the combined assessment of these tumor biomarkers, with possibly other parameters, rather than relying on a single element, can facilitate the early detection of CRC recurrence, besides their utilization as a prognostic tool and eventually during the follow-up of anticancer therapy.

This study also investigated the potential benefits of intermittent fasting in CRC patients receiving chemotherapy during the month of Ramadan. The majority of patients in the current study have reported improvement in intolerability of the chemotherapy side effects. Less than 20% of patients reported worsening nausea while fasting, but it did not affect their usual activities of daily living. This is consistent with a number of studies that have reported no significant changes during short-term fasting or even a substantial reduction in the chemotherapy-related adverse effects, particularly in hematological toxicity, GI upsets, and fatigue [22-24].

Due to the hot and dry climate, in Saudi Arabia during the month of Ramadan, dehydration and the risk of worsening renal and liver function tests are among the critical concerns of prolonged fasting periods in the current study settings. However, both eGFR and alkaline phosphatase showed no significant changes pre and post-fasting, minimizing such concerns.

Moreover, several reports have also suggested a potential improvement in the cancer therapeutic outcomes if cancer chemotherapy was combined with short-term fasting due to restriction of nutrient intake without causing weight loss [25]. Furthermore, fasting could even augment cancer immunotherapy strategies by inducing cancer-specific T-cell activation, which plausibly enhances the cancer cell-killing effect [26].

All these previous results have supported the safety and the potential benefit of intermittent fasting or short-term fasting among CRC patients during chemotherapy due to the alleviation of adverse effects and possible attribution to the profound metabolic changes in the human body that occurs during nutrient restriction such as reduction of the circulating levels of growth factors, and inflammatory cytokines that are associated with various malignancies [27,28]. This study also showed no increased toxicity of chemotherapy, as the biomarkers CEA and LDH did not increase during fasting. Moreover, as described above, it was demonstrated that fasting had no negative effects on renal function. This was confirmed by no significant changes in serum creatinine levels, and pre- and post-fasting eGFR values, which refute the concern of renal impairment during fasting.

This study is clinically important because it provides a piece of good evidence that fasting among CRC patients during Ramadan may not be detrimental in the majority of patients and can be practiced safely in patients who wish to do so. Allowing patients to practice their faith by fasting without interrupting their treatment protocols may enhance their overall well-being, and improve the doctor-patient relationship, which may positively affect their health outcomes.

Lastly, the small sample size is an inevitable limitation of the current study that may have impacted the significance and generalizability of the study results. Furthermore, it is important to report that CEA and LDH levels are not routinely measured during the adjuvant chemotherapy setting in CRC patients. Recruited patients in this study had different CRC stages, some being in the early stages (II and III), however, the majority had stage IV metastatic disease. Hence, the effects of fasting on CEA and LDH levels may perhaps be overestimated.

## Conclusions

In summary, this study supports the safety of Ramadan fasting for CRC patients receiving chemotherapy. The results of this study were also consistent with several reports that have demonstrated the safety of intermittent fasting with enhanced tolerability and possibly improved therapeutic outcomes of cancer chemotherapy. However, the levels of CEA and LDH tumor biomarkers, which are frequently associated with CRC, were not valuable in assessing the benefits of intermittent fasting during chemotherapy. Thus, further studies with a larger population and more extended follow-up periods are still necessary to evaluate the role of CEA and LDH levels as diagnostic, and prognostic tumor biomarkers or to monitor patients’ responses to anticancer therapy.

## References

[1] Lessan N, Ali T (2019). Energy metabolism and intermittent fasting: the Ramadan perspective. Nutrients.

[2] Sundfør TM, Svendsen M, Tonstad S (2018). Effect of intermittent versus continuous energy restriction on weight loss, maintenance and cardiometabolic risk: A randomized 1-year trial. Nutr Metab Cardiovasc Dis.

[3] Browning JD, Baxter J, Satapati S, Burgess SC (2012). The effect of short-term fasting on liver and skeletal muscle lipid, glucose, and energy metabolism in healthy women and men. J Lipid Res.

[4] Al-Jafar R, Zografou Themeli M, Zaman S (2021). Effect of religious fasting in Ramadan on blood pressure: results from LORANS (London Ramadan Study) and a meta-analysis. J Am Heart Assoc.

[5] Li C, Sadraie B, Steckhan N, Kessler C, Stange R, Jeitler M, Michalsen A (2017). Effects of a one-week fasting therapy in patients with type-2 diabetes mellitus and metabolic syndrome - A randomized controlled explorative study. Exp Clin Endocrinol Diabetes.

[6] Nencioni A, Caffa I, Cortellino S, Longo VD (2018). Fasting and cancer: molecular mechanisms and clinical application. Nat Rev Cancer.

[7] Almatroudi A (2020). The incidence rate of colorectal cancer in Saudi Arabia: an observational descriptive epidemiological analysis. Int J Gen Med.

[8] Sung H, Ferlay J, Siegel RL, Laversanne M, Soerjomataram I, Jemal A, Bray F (2021). Global Cancer Statistics 2020: GLOBOCAN estimates of incidence and mortality worldwide for 36 cancers in 185 countries. CA Cancer J Clin.

[9] Munker S, Gerken M, Fest P (2018). Chemotherapy for metastatic colon cancer: no effect on survival when the dose is reduced due to side effects. BMC Cancer.

[10] Campos-da-Paz M, Dórea JG, Galdino AS, Lacava ZG, de Fatima Menezes Almeida Santos M (2018). Carcinoembryonic antigen (CEA) and hepatic metastasis in colorectal cancer: update on biomarker for clinical and biotechnological approaches. Recent Pat Biotechnol.

[11] Forkasiewicz A, Dorociak M, Stach K, Szelachowski P, Tabola R, Augoff K (2020). The usefulness of lactate dehydrogenase measurements in current oncological practice. Cell Mol Biol Lett.

[12] Wei Y, Xu H, Dai J, Peng J, Wang W, Xia L, Zhou F (2018). Prognostic significance of serum lactic acid, lactate dehydrogenase, and albumin levels in patients with metastatic colorectal cancer. Biomed Res Int.

[13] Aday U, Böyük A, Akkoç H (2020). The prognostic significance of serum lactate dehydrogenase-to-albumin ratio in colorectal cancer. Ann Surg Treat Res.

[14] Lopes G, Stern MC, Temin S (2019). Early detection for colorectal cancer: ASCO Resource-Stratified Guideline. J Glob Oncol.

[15] Beets G, Sebag-Montefiore D, Andritsch E (2017). ECCO essential requirements for quality cancer care: colorectal cancer. A critical review. Crit Rev Oncol Hematol.

[16] Boonpipattanapong T, Chewatanakornkul S (2006). Preoperative carcinoembryonic antigen and albumin in predicting survival in patients with colon and rectal carcinomas. J Clin Gastroenterol.

[17] Wild N, Andres H, Rollinger W, Krause F, Dilba P, Tacke M, Karl J (2010). A combination of serum markers for the early detection of colorectal cancer. Clin Cancer Res.

[18] Chen JS, Chen KT, Fan WC, Yu JS, Chang YS, Chan EC (2010). Combined analysis of survivin autoantibody and carcinoembryonic antigen biomarkers for improved detection of colorectal cancer. Clin Chem Lab Med.

[19] Tóth K, Sipos F, Kalmár A (2012). Detection of methylated SEPT9 in plasma is a reliable screening method for both left- and right-sided colon cancers. PLoS One.

[20] Passardi A, Scarpi E, Tamberi S (2015). Impact of pre-treatment lactate dehydrogenase levels on prognosis and bevacizumab efficacy in patients with metastatic colorectal cancer. PLoS One.

[21] Marmorino F, Salvatore L, Barbara C (2017). Serum LDH predicts benefit from bevacizumab beyond progression in metastatic colorectal cancer. Br J Cancer.

[22] Dorff TB, Groshen S, Garcia A (2016). Safety and feasibility of fasting in combination with platinum-based chemotherapy. BMC Cancer.

[23] Safdie FM, Dorff T, Quinn D (2009). Fasting and cancer treatment in humans: a case series report. Aging (Albany NY).

[24] Bauersfeld SP, Kessler CS, Wischnewsky M (2018). The effects of short-term fasting on quality of life and tolerance to chemotherapy in patients with breast and ovarian cancer: a randomized cross-over pilot study. BMC Cancer.

[25] Lee C, Longo VD (2011). Fasting vs dietary restriction in cellular protection and cancer treatment: from model organisms to patients. Oncogene.

[26] Pardoll DM (2012). Immunology beats cancer: a blueprint for successful translation. Nat Immunol.

[27] de Groot S, Pijl H, van der Hoeven JJ, Kroep JR (2019). Effects of short-term fasting on cancer treatment. J Exp Clin Cancer Res.

[28] Zhang J, Deng Y, Khoo BL (2020). Fasting to enhance cancer treatment in models: the next steps. J Biomed Sci.

